# Exploring chatbot trust: Antecedents and behavioural outcomes

**DOI:** 10.1016/j.heliyon.2023.e16074

**Published:** 2023-05-06

**Authors:** Subburaj Alagarsamy, Sangeeta Mehrolia

**Affiliations:** a School of Business, Manipal Academy of Higher Education, Dubai Campus, United Arab Emirates; b School of Business and Management, Christ University, Bangalore, 560029, India

**Keywords:** Chatbot, Trust, Technology acceptance, Risk, Diffusion of innovation

## Abstract

An awareness about the antecedents and behavioural outcomes of trust in chatbots can enable service providers to design suitable marketing strategies. An online questionnaire was administered to users of four major banking chatbots (SBI Intelligent Assistant, HDFC Bank's Electronic Virtual Assistant, ICICI bank's iPal, and Axis Aha) in India. A total of 507 samples were received of which 435 were complete and subject to analysis to test the hypotheses. Based on the results, it is found that the hypothesised antecedents, except interface, design, and technology fear factors, could explain 38.6% of the variance in the banking chatbot trust. Further, in terms of behavioural outcomes chatbot trust could explain, 9.9% of the variance in customer attitude, 11.4% of the variance in behavioural intention, and 13.6% of the variance in user satisfaction. The study provides valuable insights for managers on how they can leverage chatbot trust to increase customer interaction with their brand. By proposing and testing a novel conceptual model and examining the factors that impact chatbot trust and its key outcomes, this study significantly contributes to the AI marketing literature.

## 1 Introduction

The Indian banking sector has undergone significant disruptions in recent years and witnessed several financial service innovations. Digitalisation and automation are two such innovations that Indian banks have adopted to compete with their global counterparts and offer their customers a complete range of sophisticated services, which their customers can access online 24 × 7 × 365 days. According to an IBV [1] survey, 67% of Indian banks have implemented Artificial Intelligence (AI), Big Data, and analytical tools to improve customer engagement and enhance their experience. Another innovation gaining ground is the chatbot services which banks are implementing to engage with their customers.

The chatbot is a virtual human interaction app used for customer relationship management, customer assistance, navigation, recommending products and services, grievance handling, and investment analysis [[2], [3], [4]]. Chatbots make banking activities cost-effective and reduce human effort [[5], [6], [7]].

As with other chatbots, customers remain apprehensive about the use of banking chatbots primarily because of concerns of data safety [8]. Ubiquity, personalisation, identification, and instantaneity are some factors that define the chatbot service [[8], [9], [10], [11]]. Even though India has a 624 million internet users market, the use of the internet for financial services is not routine [12]. Furthermore, chatbots are programmed to fulfil both business-to-consumer (B2C) and business-to-business (B2B) needs [8]. However, they can only respond to pre-defined questions and are therefore unable to address customer queries outside their ambit, As a result, customer dissatisfaction with AI chatbots is primarily because of the inappropriate responses they provide. Further, they cannot mimic human service agents, which in turn, leads to customer dissatisfaction [7].

Studies have also raised concerns about the potential misuse of consumer data [9]. Despite mixed views and potential growth in AI-related literature, research in this area remains limited [7]. Most research on online customer trust focuses on the general trust built between organizations and consumers over time, with many studies exploring trust in e-commerce and m-commerce [[13], [14], [15]]. However, factors influencing trust may vary across platforms and communication techniques, especially for chatbots with their unique human-like features [13]. Research on chatbot trust remains in its early stages [8], and trust in AI is an important aspect to consider [16]. Future studies involving chatbots should not overlook trust, particularly trust developed after initial interactions with new technologies [17,18]. To date, few studies have investigated predictors of trust in chatbots, highlighting the need for further exploration to create trustworthy conversational agents [8,19]. Companies view customer satisfaction and behavioural intentions as crucial outcomes of their online presence [8,13,20,21]. Many studies have argued that the level of trust in virtual technologies affects customer engagement [3,16,22]. However, previous marketing scholars have underestimated the impact of trust on customer attitude, satisfaction, and behavioural intentions in e-commerce, warranting further investigation [8,10].

There is a growing interest among both academics and practitioners in researching the use of chatbots to enhance customer-centric services for businesses. However, existing literature on this subject has often relied on disparate theories, with limited publications integrating well-established conceptual frameworks or offering comprehensive discursive contributions [23]. This study is a step to addresses this gap in the literature. It aims to identify the antecedents of trust and its relationship with different behavioural outcomes, i.e., customer attitude, customer satisfaction and behavioural intention in chatbot applications.

## 2 Theoretical background and hypotheses development

### 2.1 Chatbot trust

Studies have examined the concept of trust within the context of human interpersonal relationships with particular focus on web vendors or virtual team members in the IS field [[24], [25], [26], [27]]. Recently, there has been shift in attention to evaluating trust in technology, such as an AI-based or non-AI-based information system [5,9,16,17,20,22]. Users' trust determines the success of any IS-based service [13,21,28,29]. Prior research has claimed that “trust is a dynamic notion that grows with time, especially when customers have to overcome ambiguity before using the new technology” [6,8]. Consumer trust in new technology grows with use over a period of time. In the context of chatbots it is seen that the natural language interaction and other human-like aspects of AI chatbots provide a sense of human contact and friendliness that stimulates people's social presence, however, this is not the case of non-AI technologies [2,8]. It is worth emphasizing that technologies that foster a sense of social presence are more likely to be trusted [30]. Customers may also fail to distinguish between a chatbot and a conversational human being while interacting with a chatbot [6,9]. The human-like traits of chatbots can facilitate trust and emotional connect between the customers and the technology. Thus, to effectively implement chatbot applications banks need to understand the importance of users' trust in chatbot applications, their antecedents and behavioural outcomes. This study analyses the role of trust in chatbot acceptance and the novel features that can be incorporated in technology to build trust in the human-technology interaction.

### 2.2 Antecedents of chatbot trust

#### 2.2.1 Technology acceptance factors

Numerous academics have advocated that perceived usefulness and ease of use are the most critical components of technology acceptance and trust [28,31,32]. However, perceived enjoyment is another critical component that has not been studied to predict trust [2,33,34]. Therefore, to bridge this gap in literature, we have confined our analysis to perceived usefulness, perceived ease of use, and perceived enjoyment. Several IS theories, such as the Technology Acceptance Model (TAM) [35], Unified Theory of Acceptance and Use of Technology (UTAUT) [36], and Diffusion of Innovation theory (DOI) [37] can help explain the links between perceived ease of use, perceived usefulness, perceived enjoyment, and chatbot trust.

##### 2.2.1.1 Perceived ease of use

Perceived ease of use refers to the level at which a system runs smoothly and does not require additional skill sets, knowledge and effort from customers [32,38,39]. Increased trust is associated with an increase in the level of perceived ease of use. The TAM and UTAUT model describes the perceived ease of use as effort expectancy; therefore, studies are seen to use these two words interchangeably [24,[39], [40], [41], [42]]. According to the DOI theory, customers' behaviour with new technology is determined by their perceptions about its use. One of the technology-related facets of the DOI theory is complexity, which is the opposite of perceived ease of use [28,[43], [44], [45]]. According to Zhao et al. (2018), service providers can quickly eliminate hesitation in using Internet-based services by emphasizing perceived ease of use [46]. Thus, we hypothesise that.

H1 Perceived ease of use positively influences chatbot trust

##### 2.2.1.2 Perceived usefulness

The system's perceived usefulness feature improves when it facilitates and improvises job performance [34,39,45]. Any service that helps the customer save time and gives customised services and flexibility creates a positive perception of the service provider [24,35,39,47,48]. Previous scholarly research on m-commerce and e-commerce has highlighted this usefulness feature and how it influences customers' initial trust [13,28,32,49]. Perceived usefulness is similar to the performance expectancy of the UTAUT model [40,41] and the relative advantage of the DOI theory [50]. Users acquire trust in various IS due to their perception of these benefits [45,51]. According to previous research, users' assessment of the information system's usefulness has a favourable effect on their trust. Thus, we propose the following hypothesis.

H2 Perceived usefulness positively influences chatbot trust

##### 2.2.1.3 Perceived enjoyment

Perceived enjoyment is the level of satisfaction and happiness a platform gives during the process [2,33,34,38]. The more enjoyable an experience is, the more the customers harbour a positive intention toward using IS services [38,39]. The enjoyment construct has become equally crucial as usability and perceived ease, and plays an essential role in adopting information technology-related products and services [33,52]. Previous literature on IS and technology acceptance has emphasised that intrinsic motivation (i.e., enjoyment, fun, entertainment and playfulness) is critical for building customer trust and intention to utilize new systems and applications [36,53]. Additionally, chatbot services are viewed as a new and innovative technology that may provide customers with a sense of excitement and satisfaction through the interaction [2]. Numerous research cases investigating the customers' willingness to use IS have found a significant effect of perceived enjoyment [5,41,42]. Additionally, improving the customers' intrinsic benefits ensures that they perceive the targeted system as more valuable and trustworthy. As a result, we propose the following hypothesis.

H3 Perceived enjoyment positively influences chatbot trust

#### 2.2.2 Quality factors

A system's quality can be measured in terms of its information, system and the services offered. These characteristics affect subsequent usage or intention to use and user satisfaction [[54], [55], [56]]. Certain benefits would be realized because of system usage. Based on the DeLone and McLean Information Systems Success Model (D&M success model) [57], we have confined our analysis to the factors of information quality, service quality and interface design (system quality).

##### 2.2.2.1 Information quality

Users always look for timely, accurate, and updated information while using any virtual app and online service [29,47,58,59]. The digital platform should have inbuilt information relevance, sufficiency, timeliness, and accuracy features to generate trust in online services [55,[60], [61], [62]]. Virtual human interaction apps are usually faster than search engines and smarter in understanding user queries [2,56]. Many research studies have highlighted the importance of information quality to build trust across e-commerce, m-commerce and other virtual app-based services that different businesses adopt [13,63,64]. Information quality has been quantified by assessing an information system's output in terms of timeliness, correctness, understandability, interest, completeness, reliability, and trustworthiness [13,63]. Information quality affects user's trust in the system and their intentions towards its use, which affects the system's ability to generate benefits for both the user and the business. Additionally, studies indicate that information quality affects service quality, another significant predictor of the user. Numerous research studies have concluded that improving service quality would be difficult, if not impossible, without a high degree of information quality trust [2,58,63]. This leads us to the following hypothesis.

H4 Information quality positively influences chatbot trust

##### 2.2.2.2 Service quality

Service quality is another variable that helps to build trust and the system's reliability, responsiveness, assurance, and personalisation capability. A sense of high quality is fostered by consistent, reliable service, which encourages users to trust the system [3,65]. When service quality is timely, quick, and personalised, users perceive it positively, which helps to build trust [66,67]. Employees' competence, knowledge, civility and ability to build trusting relationships with customers are all indicators of “service quality assurance” [68,69]. If a chatbot service has the “knowledge and ability to inspire trust and confidence” in users, they will have a greater intention towards its use [63,65]. This discussion clarifies that chatbot service quality improves user trust. As a result, the following hypothesis is advanced.

H5 Service quality positively influences chatbot trust

##### 2.2.2.3 Interface design

While interaction is vital in enacting user engagement, trust is primarily based on quality value judgements about the service [15,59,70]. According to literature, smart device interactivity is a strong driver for users' favourable reactions to device usage, and as well as the evaluation of their post-use confirmation [71,72]. Interface design significantly influences user interactivity, which further influences user trust [73]. Interface design refers to the website's layout, the flow of the pages, and navigation quality [74,75]. Online service providers aim to create a user-friendly environment that reduces complexities and improves interactivity, facilitating ease of use [13,71]. Digital platforms should provide a feature of interactivity that allows the customer to create a conversation similar to a desk interaction. A well-designed user interface decreases the system's perceived complexity, simplifies navigation and engagement and instils trust in the users of the system [10,15,70,73,74]. Consequently, the following hypothesis is proposed.

H6 Interface design positively influences chatbot trust

#### 2.2.3 Risk factors

Risk factors influence the confidence of individuals in their decisions. Circumstances can be risky when the probabilities of outcomes are unknown and the result is either known or unknown [76]. Prior consumer research studies describe the perceived risk factors as the perception of ambiguity in a purchasing transaction [24,77]. This poses a ‘risk’ because users are unaware of the significance of this discrepancy. If technology fails to achieve the intended result, the user will incur a loss (financial, psychological, physical, or social). In TAM studies, perceived risk is often seen as a cause of trust or behaviour [24,76]. In this research, we have confined the risk factors to our analysis to perceived risk, privacy and security concerns and structural assurance.

##### 2.2.3.1 Perceived risk

Risk is inversely related to trust, but it is one of its essential determinants. The level of uncertainties is higher online than in traditional contexts. Hence, perceived risk is also higher in an online service context. It is vital in determining the online purchase process and customers' continuance intentions [78]. “Perceived risk is defined as subjective evaluation of incurring losses” while using banking chatbots [79]. In internet-based technologies, perceived risk is associated with perceived performance and privacy risks [19,80,81]. Users perceive chatbots as a less reliable source of communication in case of financial matters. Since information shared or demanded by users involves a high level of confidentiality, they find it risky to disclose it to AI-based applications [6,80,82]. Perceived risk by users, if elevated, can negatively impact a user's confirmation and satisfaction. Apart from this, the negative side of chatbots includes the absence of human touch and lack of empathy. Furthermore, the nature of the transaction elevates the risk of the use of these applications [28]. Perceived risk can negatively influence chatbot trust by increasing information asymmetry, decreasing the perceived usefulness and ease of use of chatbots, and increasing the perceived risks associated with using them. These effects can lead to decreased trust in social exchange processes, which are supported by theories such as the TAM [35], Protection Motivation Theory [83], and Social Exchange Theory [84]. Therefore, the following hypothesis is proposed.

H7 Perceived Risk negatively influences chatbot trust

##### 2.2.3.2 Structural assurance

Previous studies have found that structural assurance is a significant predictor of online consumer trust [13,48,61,85]. Apart from calculative-based beliefs, situational normality, and familiarity, structural assurance may be the most important predictor of a web vendor's perceived trustworthiness [24]. Many studies have found that structural assurance could boost vendor and technology trustworthiness [24,62,86]. However, some researchers argue that structural assurance is a weak predictor of a customer's trust rather than vendor reputation and site quality [59,61]. Availability of structural assurance components in building online infrastructure gives users assurance about safeguarding information security [85,87]. Structural assurance includes “promises, guarantees, regulations and contractual terms and conditions” that highlight the vendor's credibility and are directly related to trust in the system [48,61]. The Social Exchange theory suggests that trust is a social exchange process that involves assessing the benefits and risks of interacting with others [84]. Structural assurance can reduce the perceived risks associated with chatbots by providing assurance that their technology infrastructure is secure and reliable, which can increase users' trust. Thus, we propose the following hypothesis.

H8 Structural assurance positively influences chatbot trust

##### 2.2.3.3 Privacy and security concerns

While chatbots may offer various benefits to the users, general security concerns and privacy issues related to confidentiality of customers' data and sharing it with an unsolicited third-party may negatively affect their trust [15,26,88,89]. Security and privacy issues are classified into two broad categories: threats and vulnerabilities [90]. A security threat is defined as the possibility of an organization's systems and data being compromised. Computer security threats include “Spoofing, Tampering, Repudiation, Information Disclosure, Denial of Service, and Privilege Elevation” [90]. We can adopt protective mechanisms that ensure the following properties: “Authenticity, Integrity, Non-repudiation, Confidentiality, Availability, and Authorisation."

System vulnerabilities are flaws in a computer system that can be exploited by unethical hackers to traverse privilege boundaries [49,91]. The system is vulnerable if it has insecure coding, out-of-date hardware drivers, or a weak firewall, among other factors. Human error is the primary source of system vulnerabilities [26,89]. Due to this, customers are hesitant to share their full details to chatbots [9,80,82,88]. Chatbots in banks store a large amount of personal information they can be exploited for commercial purposes. Thus, the following hypothesis is proposed.

H9 Privacy and security concerns negatively influence chatbot trust

#### 2.2.4 Individual factors

IS success and human-computer interface research rely heavily on individual aspects. Thorough scrutiny shows how individuals adjust to new IT developments. The success of such improvements depends just as much on the technology as it is on the people using it. Individual aspects of consumers have been linked to the use and success of information systems in virtual environments [20,56]. Disposition of trust, technology fear and ubiquity have been considered factors influencing chatbot trust.

##### 2.2.4.1 Disposition of trust

The ‘disposition of trust’ is defined as “an individual's ability and willingness to form trust in general. This ability is a personality trait formed through an individual's lifetime” [92]. This is a personality trait that drives new decisions. Different people may vary in the time they take to make decisions under the same circumstances for the same issue. They might develop trust based on the tendency to repose their faith in humanity, personality type, experience, and background [13,44]. This disposition leads to the assumption that people, in general, can be trusted, and therefore, it has a significant and direct effect on the formation of trust, particularly in inexperienced IS users [77,92]. In the context of new IS, an individual with a higher tendency to trust others will also have higher initial trust. The lack of physical proximity in the IS setting means that disposition to trust directly impacts and affects the formation of trust [61]. Research has demonstrated that an individual's disposition of trust has a direct impact on the formation of trust [20,27,30,93]. This is supported by academic theories such as Social Learning Theory [9] and Social Identity Theory [94]. Therefore, the following hypothesis is proposed.

H10 Disposition of trust positively influences chatbot trust

##### 2.2.4.2 Technology fear

Many authors have represented the technology fear construct as technology anxiety or computer anxiety, and related it to the novelty of the technology adopted [95]. A few authors have referred to technology fear as technophobia [96]. Technophobia is the “intense fear or dislike of advanced technology or complex devices” [96]. Many users continue to be uneasy with new technologies, preferring to complete tasks using conventional methods or limiting their use of high-performance devices to basic functions, utilizing only 10–25% of their capabilities [97]. Technology fear can have a negative influence on users' trust in chatbots. The TAM proposes that users' trust in technology is influenced by their perceptions of its usefulness and ease of use. However, technology fear can increase the perceived difficulty of using chatbots, decreasing users' trust [34,98]. Similarly, the DOI theory suggests that adopting new technologies is influenced by several factors, including users' perceived risk and uncertainty [45]. Technology fear can increase users' perceived risk and uncertainty about chatbots, decreasing their trust [95]. Protection Motivation Theory proposes that users' motivation to adopt new technologies is influenced by their perceptions of the associated risks and benefits [83]. Technology fear can increase users' perceived risks associated with chatbots, leading to decreased trust. Finally, the Self-Efficacy Theory suggests that users' confidence in their ability to use new technology can influence their adoption and trust [97]. Technology fear can decrease users' confidence in their ability to use chatbots, leading to decreased trust. These discussions make clear that technology fear negatively affects user trust. The following hypothesis is therefore proposed.

H11 Technology fear negatively influences chatbot trust

##### 2.2.4.3 Ubiquity

Ubiquity refers to the convenience of customers to complete a business transaction at any point in time from anywhere [74]. Internet-based services offer ubiquity features by eliminating spatial and temporal constraints. E-commerce services, m-commerce services and chatbot applications got famous due to their inherent ubiquity characteristics [50,74,99]. Poor connectivity and service system failure may negatively affect users' experience and customer trust. Many previous studies proved that the relationship between uniquity and the users' trust is positive and direct [13,44] and this impact of uniquity on users' trust is backed by many theories. According to the Social Presence Theory, users perceive chatbots as more human-like and trustworthy when they provide a greater sense of social presence [23,100,101]. Ubiquity can increase the social presence of chatbots, making them feel more available and accessible, which can increase users' trust in them. The TAM suggests that users' trust in technology is influenced by their perceptions of its usefulness and ease of use [35]. Ubiquity can increase the perceived usefulness of chatbots by making them more available and accessible. Expectancy-Disconfirmation Model proposes that users' acceptance and satisfaction with technology are influenced by the degree to which it meets their expectations [98]. Additionally, ubiquity can increase their expectations of chatbots by making them more visible and accessible. This expectation increase can increase trust, positively influencing satisfaction by providing consistent and reliable service across multiple channels. Therefore, the following hypothesis is proposed.

H12 Ubiquity positively influences chatbot trust

### 2.3 Behavioural outcomes

According to the Theory of Reasoned Action (TRA) [102] and TAM [35] consumers' attitude affects their purchase behaviour. These theories assert that behaviour is determined by intentions, which are influenced by attitudes and subjective norms. In the context of technology acceptance and continuation, users' favourable attitudes toward a system result in favourable behavioural intentions [103]. The widespread availability of mobile technology and internet services has made it possible for virtual customers to purchase and consume services online [74,99]. However, online transactions come with inherent risks that the development of trust can mitigate [31,104]. Researchers have suggested that trust is closely linked to perceived privacy and reliability, and is critical in shaping consumers' attitudes and behaviours towards e-commerce and m-commerce services [26,105]. When consumers trust these platforms to provide secure and reliable delivery, fair pricing, and high-quality services, they are more likely to have positive attitudes and make repeat purchases [106]. Some studies suggest an indirect relationship between trust and customers' behavioural intentions to use internet-based mobile applications [107,108], while others find evidence of a direct effect of trust on customers' positive attitudes toward adopting such applications [29,106]. However, due to inconsistent findings, further research is needed to fully understand the relationship between trust and consumers' attitudes towards online transactions. Extending this logic to the chatbot context, we believe that.

H13 Trust positively influences customers' attitude

The TRA explains how a consumer's beliefs (i.e. trust) affect their purchase intentions. Numerous studies have explored the relationship between trust and customers' behavioural intentions towards internet banking [21,81]. These studies have found that trust positively impacts users, leading to an increase in their behavioural intentions. For instance, e-commerce platforms can enhance the trust of their customers by providing them with information about the security and stability of internet-based applications, which can help alleviate their concerns about the reliability of the service. This, in turn, can help to develop greater trust towards using internet-based applications. Therefore, confidence in using these applications significantly attracts more users [25,109]. Several studies show how trust positively impacts customers' behavioural intentions and the actual use of chatbots powered by artificial intelligence (AI) [4,10,28,110]. Overall, the studies suggest that trust plays a crucial role in driving behavioural intentions to use internet-based applications, and as the level of trust increases among customers, so does their positive attitude towards and intention to use these applications. The above discussions led to the following hypothesis.

H14 Trust positively influences customers' behavioural intentions

User satisfaction is one of the key concepts in both information systems and marketing research, which is often used as an indicator of the success of the information systems [54,109]. The D&M success model is a widely used framework for evaluating the success of information systems, and it includes user satisfaction as one of its dimensions [57]. User satisfaction refers to users' satisfaction with the information system and its features, including ease of use, usefulness, and reliability. It is a crucial predictor of continued system use and often used to measure system success, which is influenced by trust [111]. User satisfaction is the positive emotional response users experience on interacting with banking chatbots. It is dependent on user trust and is fulfilled when primary expectations are met [51]. Chatbot services serve as the first point of contact for users and therefore they should meet their requirements in order to foster trust and user satisfaction. Numerous studies have identified consumers' trust and satisfaction as critical factors affecting the success of partner relationships in e-commerce [13,112]. A few studies argue that lack of customer trust negatively affects consumer intentions and satisfaction [49]. Building trust is crucial for electronic commerce as it plays a significant role in fostering customer loyalty and satisfaction [113]. The cognitive dissonance theory also supports the relationship between trust and satisfaction, implying that consumers strive for consistency in their beliefs, values and perceptions [114]. Thus, when trust is high, satisfaction is expected to be high. These conclusions are considered in the following hypothesis.

H15 Trust positively influences customers' satisfaction

**Fig. 1:**
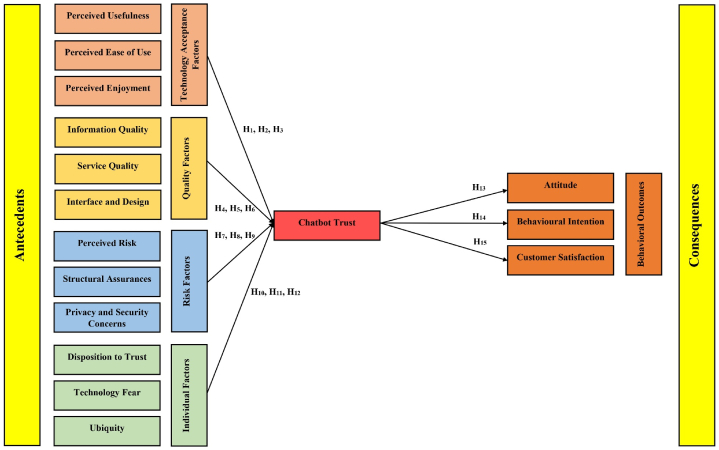
Conceptual model.

A conceptual model is proposed based on the extensive literature review presented above, as shown in Fig. 1.

## 3 Methods

### 3.1 Research paradigm

This study identifies the antecedents and consequences of chatbot trust, which can be measured using quantitative data [8,11]. This paradigm assumes that an objective reality can be measured and observed using scientific methods, such as surveys and quantitative analysis. The positivist paradigm allows statistical methods to test hypotheses and establish causal relationships between variables [115]. This approach provides a systematic and rigorous way to examine the research questions and ensures that the findings are objective and replicable [116]. Moreover, the justification of hypotheses by theories and previous studies further supports the positivist paradigm and a deductive approach, as it emphasizes using existing knowledge to explain and predict phenomena.

### 3.2 Instruments

The research instrument has six sections. All the constructs used in this research instrument are adapted from literature. The first section of the instrument consists of constructs related to chatbot technology acceptance. To measure the technology acceptance factors, three constructs are used, namely, perceived usefulness [117], perceived ease of use [118] and perceived enjoyment [52]. Secondly, to measure chatbot quality, three constructs - namely information quality [64], service quality [47], and interface & design [39,74] are adopted. The third section consists of three constructs, namely perceived risk [58,119], structural assurance [61], and privacy & security concerns [120], which measure the risk factors associated with chatbot trust. The fourth section of the instrument includes three constructs, namely disposition to trust [92], technology fear [95] and ubiquity [121], and these aim to measure individual factors that influence chatbot trust. The fifth section of the instrument consists of constructs related to individual perceptions about chatbot trust and its behavioural outcomes, such as behavioural intention [48], attitude [122] and user satisfaction [123]. All these research items are rated using a seven-point Likert scale ranging from ‘1-strongly disagree’ to ‘5-strongly agree’. The last section includes questions related to the demographics of the respondents. See appendix for complete measurement scales.

### 3.3 Sampling procedure

**Fig. 2:**
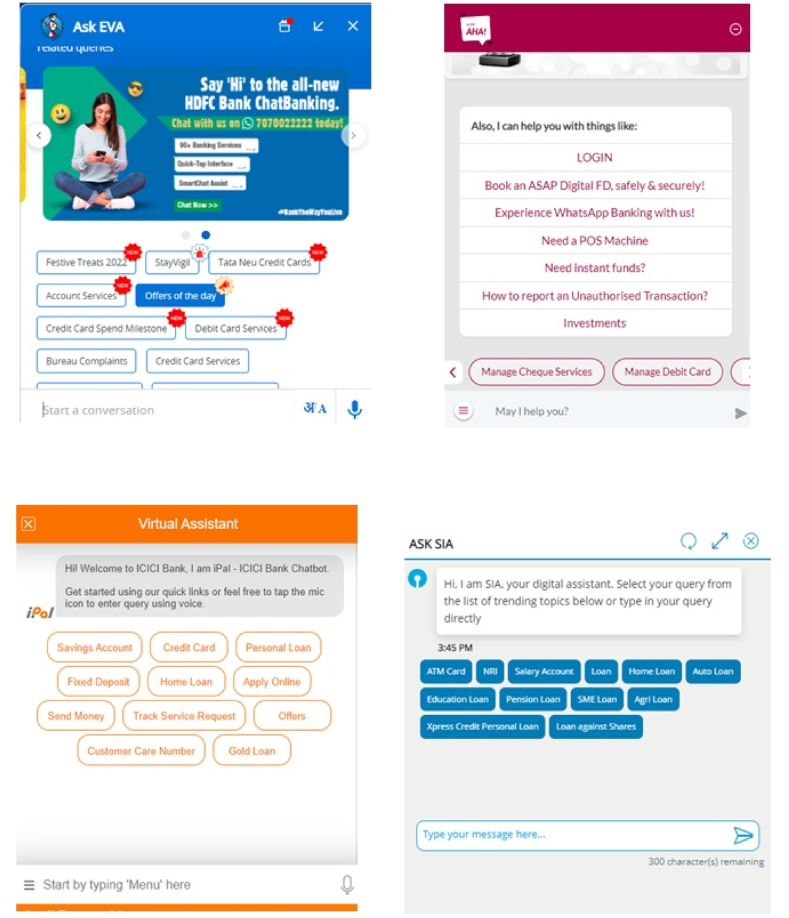
Banking chatbot screenshots.

This study collects data from users of banking chatbot services in India. For this research, four major banking chatbots were selected: SBI Intelligent Assistant (SIA), HDFC Bank's Electronic Virtual Assistant (EVA), ICICI Bank's iPal (IPAL), and Axis Aha (AHA). Screenshots of these selected banking chatbots are shown in Fig. 2. The reasons for choosing these four banking chatbot services are as follows: Asia Pacific chatbot market is growing fast. As per the Mordor Intelligence (2020) report, “the chatbot market was valued at USD 17.17 billion in 2020 and is projected to reach USD 102.29 billion by 2026, registering a CAGR of 34.75% over the forecast period, 2021–2026". Moreover, the size of the Indian chatbot market is enormous, and the need for chatbot services is increasing every year [124]. India's banking and insurance sectors are the topmost industries using AI-based chatbot services to enhance their services. The SIA, EVA, IPAL and AHA were the first to be implemented in chatbot services in early 2017 and are the most widely used in the Indian banking sector [125].

The data was collected through an online questionnaire and the respondents were selected through social media platforms, such as Facebook and LinkedIn, and also from the primary investigator's professional network contacts. This research does not include special categories, such as minors, pregnant women, differently abled individuals, prisoners or other vulnerable populations, and so it poses minimal risk to participants. The data will be used for non-sensitive purposes. Further, the data has been determined to be exempt from ethical review by the Institutional Research Conduct and Ethics Committee of Christ University, India. Respondents were requested to share the online survey link with their peers, and they were informed about the research purpose of the survey. Participation was voluntary, and the data was collected from March 2020 to April 2021. Respondents were asked to consent before participation and were free to withdraw at any time. They had to answer the survey anonymously and received no incentives for participating. We maintained confidentiality and anonymity in the research. We used a screening question " (Have you used the banking chatbot services?)" to ensure that the respondents were genuine users of chatbot services. Also, we requested the respondents to keep the banking chatbot services in mind while answering the questions. With these, we controlled their knowledge on banking chatbot services.

Moreover, ten research assistants were recruited and trained for data collection. We conducted a pilot study for the first 100 samples to determine and eliminate poorly loaded items as recommended by Hair et al. (2022). None of the research items were removed in this process. We received 507 samples, of these only 478 responses were complete, 17 were incomplete, and 12 respondents had registered their unwillingness to participate. We used Mahalanobis distance to test for multivariate outliers and identified 43 such responses which we removed from the dataset [126]. Finally, only 435 samples were considered for further analysis. The details of demographic classification are presented in Table 1.

**Table 1:** Demographic classification.

Demographics	Count	%
Age		
Less than 25 years	202	46.4
Between 26 and 35 years	92	21.1
Between 36 and 45 years	69	15.9
Between 46 and 55 years	58	13.3
Above 55 years	14	3.2
Gender		
Male	247	56.8
Female	188	43.2
Educational Qualification		
School-level	28	6.4
Bachelor's degree	275	63.2
Master's degree and above	132	30.3
Family Income (monthly)		
Less than INR. 25000	65	14.9
Between INR. 25001-50000	69	15.9
Between INR. 50001-75000	103	23.7
Between INR. 75001-100000	81	18.6
Above INR. 100000	117	26.9
Usage frequency of Banking chatbot services		
Once a month	198	45.5
Twice a month	118	27.1
Thrice a week	86	19.8
More than three times a week	33	7.6
Average time usage of Banking chatbot services		
Less than 5 min	172	39.5
Between 5 and 10 min	155	35.6
Between 10 and 20 min	86	19.8
More than 20 min	22	5.1
Total	435	100

## 4 Result

The participants' age ranged from 18 years to 67 years and averaged 32.45 years. Of these, 56.8% were male, and 43.2% were female. Also, 46.4% were less than 25 years old, 21.1% were between 26 and 35 years, 15.9% were between 36 and 45 years, 13.3% were between 46 and 55 years, and 3.2% were over 55 years. About 63.2% were undergraduates, while 30.3% were pursuing their Master's degree. Also, 6.4% of respondents had school-level qualifications, 14.9% earned less than INR 25,000, 15.9% earned between INR 25,001–50,000, 23.7% earned between INR 50,001–75,000, 18.6% earned between INR 75,001–100,000 every month. Moreover, 26.9% of the respondents earned more than INR 100,000 per month. About 45.5% of respondents used banking chatbot services once a month, 27.1% used banking chatbot services twice a month, 19.8% used them thrice a month, and only 7.6% used more than three times a month. About 39.5% used banking chatbot services for less than 5 min, 35.6% used them for 5–10 min, and 19.8% used them for 10–20 min. Only 5.1% used them for more than 20 min.

### 4.1 Common method bias

To mitigate the impact of common method bias on the empirical results of this study, we took several measures. Firstly, experts in the relevant subject and industry carefully validated the research instrument, and all items were scrutinised for ambiguity and relevance. Secondly, we maintained respondent confidentiality and anonymity to minimise the social desirability bias. Thirdly, we employed various techniques, such as counterbalancing question orders and reducing evaluation apprehension to ensure the psychological separation of the respondents [127]. Finally, we checked for method bias using two different statistical methodologies: Harman's single-factor [126] test and the Variance Inflation Factor (VIF) [128]. Results from Harman's single-factor test indicated that only 34.4% of the variance (40%) was explained by the first factor, suggesting that common method bias was not a significant issue in this study. Kock's (2015) study on common method bias in PLS-SEM concludes that “the occurrence of VIF greater than 3.3 is proposed as an indication of pathological collinearity and that a model may be contaminated by common method bias”. Table 3 shows that all the VIF values are less than 3 indicating the model can be considered free of common method bias.

### 4.2 Hypothesis testing

Hair et al. (2021) recommends that “researchers should select Partial Least Squares Structural Equation Modelling (PLS-SEM) when the analysis is concerned with testing a theoretical framework from a prediction perspective; when the structural model is complex and includes many constructs, indicators, and/or model relationships; and when distribution issues are a concern, such as lack of normality.” The study aims to examine the antecedents and behavioural outcomes of chatbot trust, and the sample size is relatively large (>400), which leads to distribution issues. Due to these reasons, the PLS-SEM is used for data analysis. SmartPLS 3 is used to test the measurement and structural models.

**Fig. 3:**
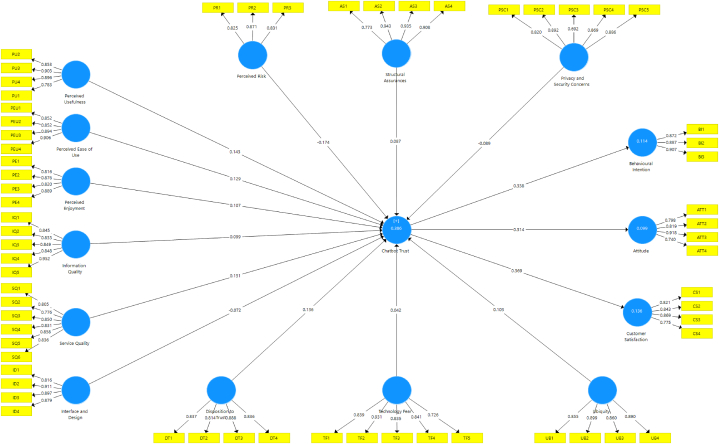
Measurement model.

The internal consistency, indicators of reliability and construct validity indicators were used to evaluate the measurement model. By examining each indicator's outer loading, which in all cases had to be more than 0.7, reliability indicators were attained. The internal consistency reliability was evaluated using Cronbach's alpha and Composite Reliability (CR). The cut-off number for both should be higher than 0.7. Convergent and discriminant validity tests can be used to determine the construct validity. If the Average Variance Extracted (AVE) is more than 0.5, convergent validity is typically regarded as satisfactory [126]. The Fornell-Larcker criterion and the Heterotrait-Monotrait ratio can determine whether a discriminant is valid (HTMT). Any indicator less than 0.4 can be removed [129]. Indicators with less than 0.4 outer loadings significantly impact AVE and CR. Fig. 3 shows that all the indicators in this study met the thresholds and were retained. Thus, the measurement model has convergent validity and internal consistency, and the results are presented in Table 2.

**Table 2:** Convergent validity and discriminant validity results.

	α	CR	AVE	1	2	3	4	5	6	7	8	9	10	11	12	13	14	15	16
1. Attitude	0.838	0.892	0.674	**0.821**															
2. Behavioural Intention	0.868	0.918	0.79	0.52 (0.615)	**0.889**														
3. Chatbot Trust	0.872	0.922	0.797	0.314 (0.363)	0.338 (0.382)	**0.893**													
4. User Satisfaction	0.847	0.897	0.685	0.064 (0.082)	0.143 (0.159)	0.369 (0.424)	**0.828**												
5. Disposition to Trust	0.866	0.908	0.712	0.148 (0.165)	0.098 (0.108)	0.207 (0.233)	0.002 (0.05)	**0.844**											
6. Information Quality	0.916	0.938	0.751	0.131 (0.146)	0.128 (0.138)	0.428 (0.478)	0.524 (0.59)	0.047 (0.066)	**0.867**										
7. Interface and Design	0.905	0.93	0.769	0.009 (0.047)	0.106 (0.115)	0.142 (0.142)	0.347 (0.382)	−0.161 (0.181)	0.268 (0.263)	**0.877**									
8. Perceived Ease of Use	0.899	0.93	0.768	0.023 (0.052)	0.072 (0.078)	0.397 (0.447)	0.456 (0.527)	0.049 (0.055)	0.401 (0.442)	0.164 (0.159)	**0.876**								
9. Perceived Enjoyment	0.872	0.913	0.724	0.016 (0.076)	0.09 (0.101)	0.429 (0.492)	0.574 (0.665)	0.028 (0.055)	0.588 (0.656)	0.235 (0.247)	0.441 (0.498)	**0.851**							
10. Perceived Risk	0.797	0.88	0.71	−0.128 (0.151)	−0.171 (0.199)	−0.381 (0.453)	−0.358 (0.433)	−0.077 (0.095)	−0.355 (0.409)	−0.36 (0.418)	−0.227 (0.264)	−0.325 (0.384)	**0.843**						
11. Perceived Usefulness	0.882	0.919	0.739	0.097 (0.111)	0.131 (0.142)	0.422 (0.48)	0.579 (0.667)	0.029 (0.049)	0.436 (0.48)	0.224 (0.229)	0.556 (0.621)	0.475 (0.54)	−0.288 (0.34)	**0.86**					
12. Privacy & Security Concerns	0.889	0.919	0.697	−0.032 (0.043)	−0.116 (0.128)	−0.23 (0.261)	−0.216 (0.245)	0.144 (0.163)	−0.222 (0.248)	−0.333 (0.363)	−0.171 (0.192)	−0.245 (0.279)	0.285 (0.339)	−0.252 (0.285)	**0.835**				
13. Service Quality	0.907	0.928	0.683	0.035 (0.079)	0.042 (0.047)	0.453 (0.506)	0.521 (0.586)	−0.007 (0.031)	0.66 (0.722)	0.228 (0.225)	0.46 (0.507)	0.615 (0.689)	−0.31 (0.361)	0.468 (0.521)	−0.267 (0.298)	**0.826**			
14. Structural Assurances	0.915	0.94	0.796	0.003 (0.048)	0.117 (0.133)	0.153 (0.163)	0.201 (0.214)	−0.066 (0.073)	0.089 (0.092)	0.354 (0.383)	0.038 (0.06)	0.1 (0.108)	−0.197 (0.22)	0.163 (0.169)	−0.091 (0.094)	0.15 (0.15)	**0.892**		
15. Technology Fear	0.891	0.921	0.701	−0.041 (0.055)	−0.142 (0.159)	−0.299 (0.339)	−0.492 (0.559)	−0.063 (0.07)	−0.476 (0.523)	−0.202 (0.209)	−0.38 (0.423)	−0.465 (0.525)	0.256 (0.295)	−0.562 (0.629)	0.129 (0.144)	−0.39 (0.43)	−0.115 (0.124)	**0.837**	
16. Ubiquity	0.901	0.929	0.767	0.227 (0.258)	0.191 (0.217)	0.13 (0.141)	−0.082 (0.096)	0.489 (0.54)	−0.029 (0.05)	−0.171 (0.174)	−0.04 (0.059)	−0.065 (0.072)	−0.041 (0.065)	−0.069 (0.075)	0.136 (0.149)	−0.113 (0.125)	−0.068 (0.076)	0.028 (0.04)	**0.876**

Note: Diagonal value shows the square root of AVE, and values inside the parenthesis represent the HTMT values; α- Cronbach Alpha, CR- Composite Reliability; AVE- Average Variance Extracted.

The Fornell-Larcker criterion was then assessed by comparing construct AVE values with shared variances. The degree of shared variance between the model's latent variables is often evaluated using this criterion. The variables are discriminatory when the AVE values are greater than the shared variance values [130,131]. The square root of AVE was higher than the inter-constructed correlations [132], supporting the discriminant validity of the constructs, as shown in Table 2. The HTMT criterion outperforms classic approaches to discriminant validity assessment in detecting a lack of discriminant validity [133]. The HTMT outcome is displayed in Table 2. All results fall below 0.90 and are within the acceptable threshold levels, disproving the concerns about discriminant validity.

This research aims to examine antecedents and behavioural outcomes of chatbot trust. Four steps were followed to test the hypotheses using structural modelling. The first step used the variance inflation factor to test the multicollinearity issues. In this structural model, technology acceptance, risk, quality, and individual factors were the independent variables; individual opinion about chatbot trust, behavioural intention, attitude towards chatbots, and user satisfaction were the dependent variables. A VIF greater than 5 indicates a potential collinearity issue in the model [134]. Table 3 shows that the retrieved VIF and its values were within the accepted threshold range, indicating no collinearity issues in the data.

The second step tested the significance of the independent and dependent constructs' path coefficient (β). The path coefficients are shown in Table 3. The structural analysis concludes that all three technology acceptance factors significantly and positively impact chatbot trust. Among those three technology acceptance factors, perceived usefulness (β = 0.143; p < 0.01) construct is the most significant predictor of chatbot trust, and not perceived ease of use (β = 0.129; p < 0.01) or perceived enjoyment (β = 0.107; p < 0.05). These finding support H_1_, H_2,_ and H_3_. In quality factors, service (β = 0.143; p < 0.01) and information quality (β = 0.099; p < 0.01) are the positive and significant predictors while interface and design (β = −0.072; p = 0.097) is not a significant predictor of chatbot trust. Thus, H_4_ is supported while H_5_ and H_6_ are not supported. In risk factors, the structural assurance factor (β = 0.087; p = 0.075) does not significantly influence chatbot trust. Perceived risk (β = −0.174; p < 0.01) and privacy and security concerns (β = −0.089; p < 0.05) factors significantly and negatively influence chatbot trust. Thus, H_7_ is supported, while H_9_ and H_8_ are not supported. Among these three risk factors, perceived risk is the most significant predictor of chatbot trust. In individual factors, disposition to trust (β = 0.136; p < 0.01) and ubiquity (β = 0.103; p < 0.01) are the positive and significant predictors, but fear of technology (β = 0.151; p = 0.384) is not a significant predictor of chatbot trust. Thus, H_10_ is supported, while H_12_ and H_11_ are not supported. These findings clarify that technology acceptance, individual, quality and quality factors are considered potential antecedents of chatbot trust. The structural model concludes that chatbot trust positively influences all three behavioural outcomes: attitude to use chatbots, behavioural intentions and user satisfaction. Among these three chatbot behavioural outcomes, the impact of a chatbot on user satisfaction (β = 0.369; p < 0.001) is stronger than the attitude to use chatbots (β = 0.314; p < 0.001) and behavioural intentions (β = 0.338; p < 0.001). Thus, H_13_, H_14,_ and H_15_ are supported. The results are presented in Fig. 3.

In the third step, the model's predictive accuracy was tested. R^2^ was used to assess “the level of the variance in the dependent variable predictable from the independent variables”. Hair et al. (2021) recommends “the acceptable R^2^ values as 0.190 weak, 0.333 moderate, and 0.670 as substantial”. As seen in Table 3, 38.6% of the variation in the chatbot trust is explained by four potential antecedents (technology acceptance factors, individual factors, quality factors and quality factors) which are moderate, while 9.9% of the variation in customer attitude towards chatbot usage is explained by chatbot trust. Also, 11.4% of the variation in user's behavioural intention towards chatbots is explained by chatbot trust. Finally, 13.6% of the variation in user satisfaction is explained by chatbot trust. These three relations are predicted weakly.

**Table 3:** Hypothesis testing.

Structural link	Beta	T-value	p-value	VIF value	Result	R^2^	Q^2^
Perceived Ease of Use → Chatbot Trust	0.129	3.201	0.001**	1.607	H_1_ Supported	0.386	0.086
Perceived Enjoyment → Chatbot Trust	0.107	2.191	0.029*	1.964	H_2_ Supported		
Perceived Usefulness → Chatbot Trust	0.143	3.004	0.003**	1.983	H_3_ Supported		
Service Quality → Chatbot Trust	0.151	2.685	0.007**	2.243	H_4_ Supported		
Information Quality → Chatbot Trust	0.099	2.069	0.039*	2.160	H_5_ Supported		
Interface and Design → Chatbot Trust	−0.072	1.660	0.097	1.441	H_6_ Not Supported		
Perceived Risk → Chatbot Trust	−0.174	4.121	0.000**	1.354	H_7_ Supported		
Structural Assurances → Chatbot Trust	0.087	2.407	0.075	1.186	H_8_ Not Supported		
Privacy and Security Concerns → Chatbot Trust	−0.089	2.660	0.016*	1.251	H_9_ Supported		
Disposition to Trust → Chatbot Trust	0.136	3.316	0.001*	1.370	H_10_ Supported		
Technology Fear → Chatbot Trust	0.042	0.871	0.384	1.676	H_11_ Not Supported		
Ubiquity → Chatbot Trust	0.103	2.744	0.006**	1.358	H_12_ Supported		
Chatbot Trust → Attitude	0.314	6.737	0.000**	1.000	H_13_ Supported	0.099	0.061
Chatbot Trust → Behavioural Intention	0.338	8.573	0.000**	1.000	H_14_ Supported	0.114	0.083
Chatbot Trust → User Satisfaction	0.369	7.756	0.000**	1.000	H_15_ Supported	0.136	0.282

**p < 0.01; *p < 0.05.

In the fourth step, the predictive relevance of the model (Q^2^) was measured using the “blindfolding technique”. Predictive relevance relates to the “accurate prediction of the data points of indicators in reflective measurement models of endogenous constructs and endogenous single-item constructs” [129]. “Q^2^ values should ideally be larger than 0 (Q^2^ > 0) to have predictive relevance”. Accordingly, all Q^2^ are larger than zero, suggesting that our model has considerable predictive power (see Table 3). However, the predictive relevance is significantly low for most of the structural links.

Finally, the structural model fit was measured using its predictive power. Model fit measures report the fitness that best represents the underlying theoretical model's data. Standardised Root Mean Square Residual (SRMR) was used to assess the model fit in PLS-SEM. The current SRMR = 0.042, which is less than 0.08, is the cut-off value [129].

## 5 Discussions

This study aims to understand the antecedents and behavioural outcomes of chatbot trust, a subject which has received very little research attention. However, few researchers have attempted to examine antecedents and behavioural outcomes of trust in e-commerce [13,111], yet, to date, no empirical research is available in the Indian context. Many studies argue that there is a lack of trust in chatbots, however, this is not tested in any of the studies [10,16,66], resulting in a gap in literature.

The results of the structural model indicate that all the hypothesised antecedents, except interface, design and technology fear factors explain 38.6% of the variance in the banking chatbot trusts. The findings reveal that all the behavioural outcomes have a significant relationship with trust in banking chatbots, and strength is considered moderate. Moreover, 9.9% of the variance in customer attitude, 11.4% of the variance in behavioural intention, and 13.6% of the variance in user satisfaction are explained by chatbot trust, and the effect strength is weak.

Hypotheses H_1_, H_2_, and H_3_ are supported as technology acceptance factors, namely “perceived enjoyment, usefulness, and ease of use”, have a strong positive impact on chatbot trust. The current results are in line with existing studies [13,32,38,40]. For example, Rouibah et al. (2016) find that perceived enjoyment positively influences consumer trust in the context of online payment systems in Arabic countries. Mostafa & Kasamani (2021) find that perceived usefulness and ease of use are significant predictors of initial chatbot trust. A possible explanation for these results may be that chatbots provide customized and flexible services to users, saving time and enhancing user trust. Also, chatbot systems provide all essential customer services on their front page, which reduces the effort required by the user and enhances enjoyment [34].

In relationship marketing, mainly information systems-based relationship marketing quality factors are considered one of the main antecedents of user trust [13]. Hypotheses H_4_ and H_5_ proposed that quality factors, namely information and service quality, have a significant positive relationship with chatbot trust. The results are consistent with the previous information system-based studies [31,50,67,73,75]. However, this study's findings contradictprevious studies [13,70,75], suggesting that the information system's interface and design don't influence chatbot trust (H_6_ is not supported). A possible explanation for this might be that the user interface of Indian banking chatbots is simple and free from additional effort at the users' level (see Fig. 2). Users do not seem to pay much importance to the chatbot's design and user interface compared to other aspects, such as quality of service and technology.

Next, we tested the impact of risk factors (perceived risk, structural assurances, and privacy and security concerns) on chatbot trust. Hypotheses H_7_ and H_9_ show that perceived risk and chatbot privacy and security concerns negatively influence user trust in chatbots. For example, Følstad et al. (2018) and Nordheim et al. (2019) conclude that there is a relationship between perceived risk and chatbot trust. Ischen et al. (2020) and Przegalinska et al. (2019) also conclude that privacy and security concerns negatively affect chatbot trust. Chatbots may pose a security risk because they use user data and can potentially “learn” from it [15,91]. This outcome may be explained by the fact that most users are unaware of how their sensitive personal information is handled, used, stored, or even shared. H_8_ is insignificant and concludes that structural assurance of chatbot does not influence the chatbot user trust, which is inconsistent with previous studies [13,79,85]. This difference could be because people do not know about the “institution-based mechanism” that gives guarantees about privacy and information protection. Indian banks do not have clear structural assurance (policies, guarantees, and regulations) that signal the banks' credibility and help to build trust in chatbots.

Individual factors, such as ubiquity (H_10_) and disposition to trust (H_12_) positively impact chatbot trust, and the results are consistent with several studies [13,20,62,99]. There are several possible explanations for these results. The ubiquitous nature of chatbots allows users to access and use real-time information wherever they are [13,45,53]. Consequently, the increased personalisation through the ubiquity of chatbot services allows users to engage in e-bank services whenever and wherever they choose. Several researchers in the information system domain have found that an individual's disposition to trust directly affects trust formation [13,20,62], in line with our results. The bank's brand image may explain that this result creates a disposition to trust chatbots, even though users possess inadequate knowledge about a chatbot or have no prior interaction. A Følstad et al. (2018) find that the brand of the chatbot host affects users' chatbot trust.

In contrast to earlier findings [[95], [96], [97],^103^], fear of technology does not significantly influence chatbot trust (H_11_). The Digital India movement encouraged many Indian consumers to start using digital payments. In India, Unified Payment Interfaces (UPI) providers recorded 2.8 billion digital payments worth more than 5 trillion Indian rupees in June 2021. The fear of using e-wallets (technology) is reducing and users' trust in e-payments is increasing. Similar effects might be a possible explanation for this inconsistent result.

According to the research, chatbot trust has a significant positive impact on user satisfaction, attitude, and behavioural intention supporting H_13_–H_15_. Previous investigations have found similar results [8,13,20,23,25,104,110]. This outcome could be explained in several ways. In the context of information systems, higher levels of user trust lead to more positive attitudes [29,[105], [106], [107], [108],^112^]. While assessing the level of satisfaction among users, their trust in banks plays a positive role. Users who trust banking chatbots continue to utilize them because they feel that banks would not exhibit any opportunistic behaviour [4,104,112].

### 5.1 Theoretical implications

The study has several theoretical and managerial implications. We have attempted to provide an overarching model that takes cognizance of various factors, such as technology, quality, risk and individual characteristics that significantly influence chatbot trust, contributing to the development of positive attitudes toward banking chatbots, and increased satisfaction and behavioural intention to use chatbots. Though trust and behavioural intention have been explored in most studies, research is scant on how these aspects might be integrated into chatbot adoption and use.

Chatbot services are a new technical interface that enhances transactions and helps to develop long-term connections with customers, assisting banks and other service-related sectors to increase user trust in chatbots. Users' trust can be linked to their expectations of how well they would perform the services. A chatbot's service attributes may also be essential in building trust perception by strengthening the experiencing features. Customers' views and opinions regarding chatbots are influenced by the level of trust they have in chatbots.

Next, the findings are classified according to users' attitudes, satisfaction, and behavioural intention to use chatbots. An earlier study has focused on a few behavioural outcomes. However, the current research has identified multiple behavioural outcomes. We find that exposure to and use of chatbots may result in implicit and explicit behaviour. This suggests that while specific behavioural results may be internalized as attitude others may visibly affect behaviour. Certain outcome variables, such as satisfaction and attitude, may have a long-term effect on consumers' behaviour, whereas behavioural intention may manifest as user behaviour. The study is important because it posits and implies that outcomes can be quantified in terms of several determinants, such as technological factors, risk, quality, and individual variables that increase the possibility that users will use chatbots in future. It establishes a unified framework for numerous features and emphasizes their application in the context of chatbot use.

Finally, these study hypotheses were based on various theoretical models, including “The Technology Adoption Model, Theory of Reasoned Action, Theory of Planned Behaviour, and Unified Theory of Acceptance and Use of Technology, Diffusion of Innovation Theory, D&M success model, Social Presence Theory, Expectancy-Disconfirmation Model, Self-Efficacy Theory, Protection Motivation Theory, Social Learning Theory, Social Exchange and Social Identity Theory ". We integrated these theoretical models to empirically study the antecedents and behavioural outcomes of the initial chatbot trust. Overall, combining multiple theories and creating a new conceptual model can help researchers generate new insights and knowledge that can benefit both the academic community and practitioners in the field. This research confirms the usefulness of the models and theories mentioned above for explaining chatbot adoption and use.

This study contributes to the literature on artificial intelligence, specifically AI chatbots, which have a revolutionary impact on marketing research. Enriching the literature on chatbots is a new influential strategy that can drastically transform how online-based companies engage with and sell to their customers. Finally, scholars have paid limited attention to the effect of trust on users' positive attitudes in the context of virtual marketing. Thus, this study adds to the body of knowledge by demonstrating a substantial positive relationship between trust and customers' attitudes in the settings of chatbots, emphasizing the critical nature of trust in virtual technologies.

### 5.2 Managerial implications

The findings of this study offer crucial insights for marketing managers working to promote their organizations, particularly in the online arena. This could be achieved by triggering four critical dimensions: technology, quality, risk, and individual aspects of chatbots. It is essential to understand that technology acceptance is one of the crucial steps in creating user trust. Therefore, it is critical to improving user acceptance by explaining the benefits and convenience of chatbot usage. Furthermore, banks should concentrate more on the development stage to create user-friendly and more enjoyable chatbots, which can provide an excellent experience for the user. To improve user acceptability, language and culture, chatbots need to be appropriately modified.

The model shows that the quality of chatbot service is another important aspect that increases trust. It can be used as a powerful driver of value creation. To improve the quality of chatbots, the design process must consider their ability to give valuable answers and solve users' problems. Lastly, while design and interface are not the main reasons why people use chatbots, they are still essential and should be given due consideration. Building and creating chatbots that meet customer service requirements can help to strengthen customer relationships.

Banks should also let their customers know that using chatbots is safe, secure, and do not pose any risks. Customers' worries about privacy and perceived risks make them less likely to trust chatbots. Even though structural assurances are not a reliable predictor of chatbot trust, they still need to be considered because the success of chatbots hinges on the banks' ability to persuade the customer to trust them and, as a result, leverage them for customer service. Many information systems emphasise the provision of effective customer service and designing aspects of their system rather than structural assurance. Structural assurance gets less attention because many customer service policies are either limited in content or are presented in standard form. As a result, banks must emphasise their customer service procedures, which will persuade customers that the bank is trustworthy.

By increasing the perceived level of the brand image through effective brand communication, banks will gain customers' goodwill, which creates a disposition of trust towards bank services, including chatbots. Banks should use mass media advertising, in-bank experience centers and tutorial videos on social media to teach their users how to use chatbot services. This will ensure that users are well-versed in their features and usage throughout, reducing technology anxiety and fear. A successful chat could significantly improve customers' experience and strengthen the bank's relationship with the customer. Additionally, banks' customer service expenses may be reduced because chatbots are supposed to provide human-like assistance to their users over time. As a result, chatbots may make a favourable impact on bank’ profits.

## 6 Limitations and future scope

This study has many limitations, which future researchers can address. The first limitation is the sample respondents. We have taken only respondents who had previously interacted with banking chatbots. Hence, future researchers can compare the difference by including other respondents such as less digital literacy. Next, this research model was developed based on various academic theories and frameworks. In addition to the constructs analyzed, other constructs such as structural assurances, technology fear, ubiquity, disposition of trust, and perceived enjoyment may also play important roles in predicting chatbot trust. Thus, researchers should further consider the role of technology optimism, novelty-seeking behaviour, and technological self-efficacy in adopting new methods. Next, generalising this result to other countries is limited because the popularity of chatbots may vary across different countries and cultures, and the present study is cross-sectional. Furthermore, customers' judgments of chatbots tend to change over time, particularly as it is in its infancy in India. It is recommended that there should be longitudinal research that can compare the results in multiple phases (initial and subsequent chatbot usage phase) and replicate the study in multi-cultural setups. In this study, no moderators were used. In future, demographic variables can be used as a potential moderator, influencing chatbot trust. Most information system-based studies concentrate on the positive side, and minimal studies investigate the dark side of information systems. Hence, it is recommended that future research should explore the dark side of AIs and chatbots in marketing.

## Author contribution statement

Subburaj Alagarsamy: Conceived and designed the experiments; Performed the experiments; Analyzed and interpreted the data; Wrote the paper.

Sangeeta Mehrolia: Performed the experiments; Contributed reagents, materials, analysis tools or data; Wrote the paper.

## Data availability statement

Data will be made available on request.

## Additional information

Supplementary content related to this article has been published online at [URL].

## Declaration of competing interest

The authors declare that they have no known competing financial interests or personal relationships that could have appeared to influence the work reported in this paper
