# Supplementary material for: Exploring chatbot trust: Antecedents and behavioural outcomes
Source: Heliyon. 2023 May 6;9(5):e16074. doi: 10.1016/j.heliyon.2023.e16074 (PMC10189503; doi:10.1016/j.heliyon.2023.e16074)
Supplement: Multimedia component 1 [file mmc1.docx]

**Appendix A**

**Technology Acceptance Factors**

**Perceived usefulness (Oghuma, Libaque-Saenz, Wong & Chang, 2016)**

1. I find the chatbot useful in my daily life
2. Using the chatbot helps me to accomplish things more quickly
3. Using chatbot increases my productivity
4. Using the chatbot helps me to perform many things more conveniently

**Perceived ease of use (Liao, Chen & Yen, 2007)**

1. My interaction with the chatbot services is clear and understandable
2. Interaction with the chatbot does not require a lot of my mental effort
3. It is easier to use the chatbot to find products that I want to buy
4. I find the chatbot to be easy to use

**Perceived enjoyment (Lee & Choi, 2017)**

1. I enjoy a conversation with the chatbot
2. It is fun and pleasant to share a conversation with the chatbot
3. The conversation with the chatbot is exciting
4. I enjoy choosing products more if the chatbot recommends them than if I choose them myself

**Quality Factors**

**Information quality (Teo et al, 2008)**

1. Information provided by this chatbot is in a useful format
2. Information provided by this chatbot is clear
3. Information provided by this chatbot is accurate
4. Information provided by this chatbot is up-to-date
5. Information provided by this chatbot is reliable

**Service quality (Roca et al, 2006)**

1. The chatbot has a modern-looking interface
2. The chatbot provides the right solution to my request
3. The chatbot gives me a prompt response
4. The chatbot has visually appealing materials
5. The chatbot gives me individual attention
6. The chatbot has an excellent interface to communicate my needs

**Interface and Design (Li, & Yeh, 2010; Nikou & Economides, 2017)**

1. The chatbot's screen design (ie, colors, boxes, navigation bars, etc) is attractive
2. The chatbot looks professionally designed
3. Navigation through the chatbot is easy
4. I like the interactivity the chatbot provides me

**Risk Factors**

**Perceived risk (Lai-Ming Tam, 2012;** **Trivedi, 2019)**

1. I perceived the chatbots service as risky
2. I perceived that while using the chatbots, there was a chance that something could go wrong in the outcome
3. I perceived that the chatbots service outcome and effect were difficult to predict

**Structural Assurances (McKnight, Choudhury & Kacmar, 2002)**

1. The chatbot services have enough safeguards to make me feel comfortable using them to transact personal business
2. I feel assured that legal and technological structures adequately protect me from problems with the chatbot services
3. I feel confident that encryption and other technological advances on chatbot services make it safe for me to do business there
4. In general, the chatbot services is now a robust and safe environment in which to transact business

**Privacy and security concerns (Son & Kim, 2008)**

1. I am concerned about the security of personal information exchange on the chatbot
2. I am concerned that my personal information may be shared with business without my consent due to usage of chatbot
3. I am concerned that the information I disclosed to this chatbot may be misused
4. I am concerned about providing personal information to this chatbot because it could be used in a way, I did not foresee
5. I am worried about the security of financial transactions carried out on chatbot

**Individual Factors**

**Disposition to trust (Lee and Turban, 2001)**

1. It is easy for me to trust a person/thing
2. My tendency to trust a person/thing is high
3. I tend to trust a person/thing even though I have little knowledge of it
4. Trusting someone or something is not difficult

**Technology fear (Cabrera-Sánchez, Villarejo-Ramos, Liébana-Cabanillas & Shaikh, 2021)**

1. I hesitate to use chatbot because I am afraid of making mistakes that I cannot correct
2. I dislike working with chatbot that are smarter than me
3. I am afraid of working with chatbot
4. I feel distressed when working with chatbot
5. I feel unsure of my ability to understand chatbot

**Ubiquity (Compeau & Higgins, 1995)**

1. I could complete a job or task using a chatbot
2. I could complete a job or task using a chatbot if someone showed me how to do it first
3. I was fully able to use a chatbot before I began using chatbot
4. I can navigate easily through a chatbot to find any information I need

**Trust– Gu et al (2009)**

1. I believe the chatbot is trustworthy
2. I believe the chatbot keeps its promises and commitments
3. I believe the chatbot considers customers' profit as top priority

**Behavioral Outcomes**

**Behavioural intention (Gu Lee & Suh, 2009)**

1. I intend to use chatbot continuously in the future
2. I will recommend others to use chatbot services
3. I will frequently use chatbot in the future

**Attitude (Dabholkar & Bagozzi, 2002)**

1. I like receiving services through chatbot
2. I think it is all right to receive services through chatbot
3. I think receiving services through chatbot is good
4. Receiving services through chatbot is comfortable

**Customer satisfaction (Bogicevic, Bujisic, Bilgihan, Yang, & Cobanoglu, 2017)**

1. I am happy with the experiences I have had with chatbot
2. I have been satisfied with my experiences with chatbot
3. My choice of chatbot selection was a wise one
4. I think that I did the right thing when I chose chatbot instead of traditional services
